# Circulating Tumor Cells as a Tool for Assessing Tumor Heterogeneity

**DOI:** 10.7150/thno.34337

**Published:** 2019-06-19

**Authors:** Marta Tellez-Gabriel, Marie-Françoise Heymann, Dominique Heymann

**Affiliations:** 1 RNA and molecular pathology (RAMP) research group. Department of Medical Biology. The Artic University of Norway (Tromso), Norway.; 2 INSERM, European Associated Laboratory “Sarcoma Research Unit”, Department of Oncology and Metabolism, Medical School, University of Sheffield, United Kingdom.; 3 INSERM, Institut de Cancérologie de l'Ouest, LabCT, U1232, CRCINA, Université de Nantes, Université d'Angers, 44805 Saint Herblain cedex, France.; 4 Institut de Cancérologie de l'Ouest, “Tumor Heterogeneity and Precision Medicine”, 44805 Saint Herblain cedex, France.

**Keywords:** single cell, circulating tumor cell, precision medicine, tumor heterogeneity, omic technologies

## Abstract

Tumor heterogeneity is the major cause of failure in cancer prognosis and prediction. Accurately detecting heterogeneity for the development of biomarkers and the detection of the clones resistant to therapy is one of the main goals of contemporary medicine. Metastases belong to the natural history of cancer. The present review gives an overview on the origin of tumor heterogeneity. Recent progress has made it possible to isolate and characterize circulating tumor cells (CTCs), which are the drivers of the disease between the primary sites and metastatic foci. The most recent methods for characterizing CTCs are summarized and we discuss the power of CTC profiling for analyzing tumor heterogeneity in early and advanced diseases.

## Introduction

**Figure 1:**
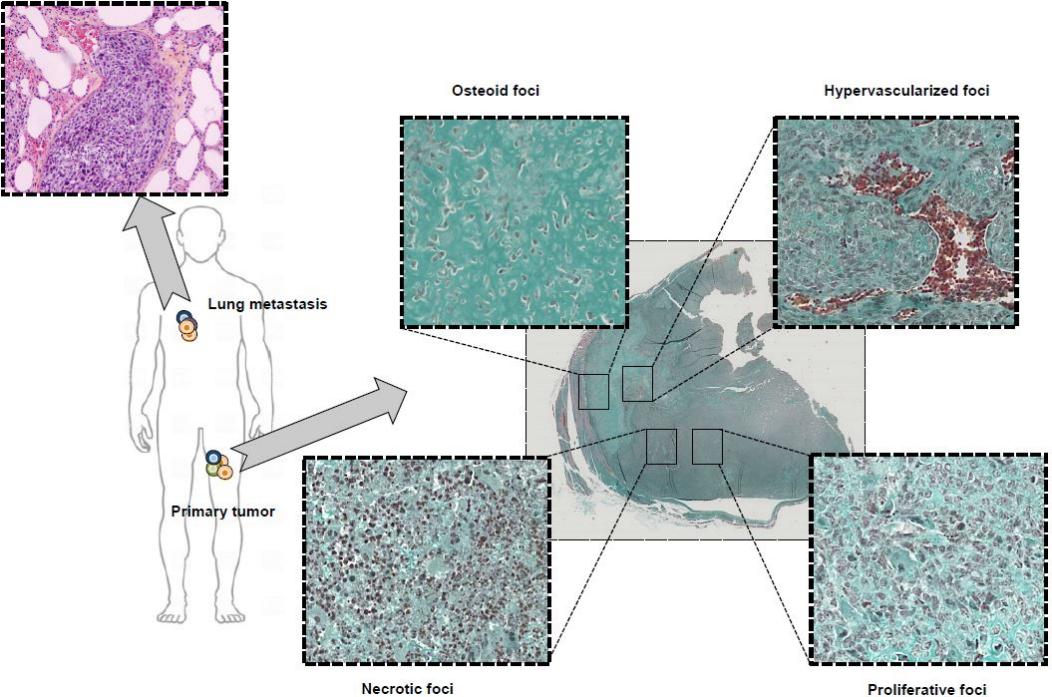
** Typical microscopic observation of tumor heterogeneity.** Osteosarcoma is a rare form of bone cancer mainly affecting adolescents and young adults. Osteosarcoma is a perfect illustration of highly heterogeneous tumors with multiple, diverse histological areas in a same tumor mass including osteoid, hypervascularized, proliferative and necrotic foci. In addition, associated lung metastases exhibit a histological morphology different from the primary tumor highlighting the contribution and effect played by the pressure of the local micro-environment on tumor heterogeneity.

Anyone who has observed a tumor section under a microscope is familiar with the significant heterogeneity of the tumor mass. Within a single tumor mass, several foci with various, specific histological features can cohabit (Figure 1). In addition to their diverse morphology, cancer cells exhibit considerable heterogeneity in terms of genetic profile, gene expression, metabolic property, motility, proliferation and metastatic potential 1. As proposed by S. Paget with the “seed and soil” theory, a symbiosis is established between cancer cells and their local micro-environment, defining the notion of tumor niche favorable for tumor growth 1. Cancer cells regulate their environment by direct cellular contacts 1 or by secreting soluble extracellular vesicles 1. In return, the micro-environment impacts the differentiation, proliferation and death of cancer cells, as well as contributing to a selection process leading to drug resistance, cell dormancy 1 and to an immune-tolerant environment 1. Cancer cells are then functionally entangled with numerous other cell types (e.g. stromal cells, endothelial cells, immune infiltrate) that enrich the heterogeneous character of tumor tissues. Consequently, tumor heterogeneity is not a fixed state but should be considered as a dynamic ecosystem that evolves as the tumor progresses and is strongly modulated under therapeutic pressure. Tumor heterogeneity is subject to major spatial and temporal modulations that can impair sustained therapeutic response and drug sensitivity.

The evolution in good clinical practices (e.g. needle biopsies) has recently led to a significant decrease in the amount of biological material available for molecular investigations. A biopsy cannot reflect and illustrate spatial tumor heterogeneity and should be considered as a partial photograph of the tumor mass at a given time. The follow-up of the dynamic process requires repeated sampling carried out using low invasive methods and should be as representative as possible of the tumor mass. Repeated biopsies are ethically non-acceptable and sometimes unrealistic for high-risk localizations of metastatic foci (e.g. the spine). The establishment of metastatic disease is directly related to the migration of cancer cells called circulating tumor cells (CTCs) into the blood and/or lymphatic circulation, plus their ability to reach distant organs 1. However, although CTCs are theoretically easily accessible, their low number makes it challenging to isolate and characterize them. The last decade has seen a surge in the new devices available for isolating CTCs, and specific downstream analysis workflows have been proposed in order to evaluate their biological value (e.g. therapeutic response, drug resistance, reflection of the tumor heterogeneity). The present review will give a brief overview of tumor heterogeneity: its origin, biological and clinical impacts and the methods for assessing it. We will put specific focus on the recent methods developed for isolating single cells and how these single cells can help to decipher this heterogeneity. We will also discuss how better characterization of single cells may orient future clinical decisions.

## Tumor heterogeneity: origins and enrichment

### Origin of cancer cell heterogeneity: from monoclonal to polyclonal disease

**Figure 2:**
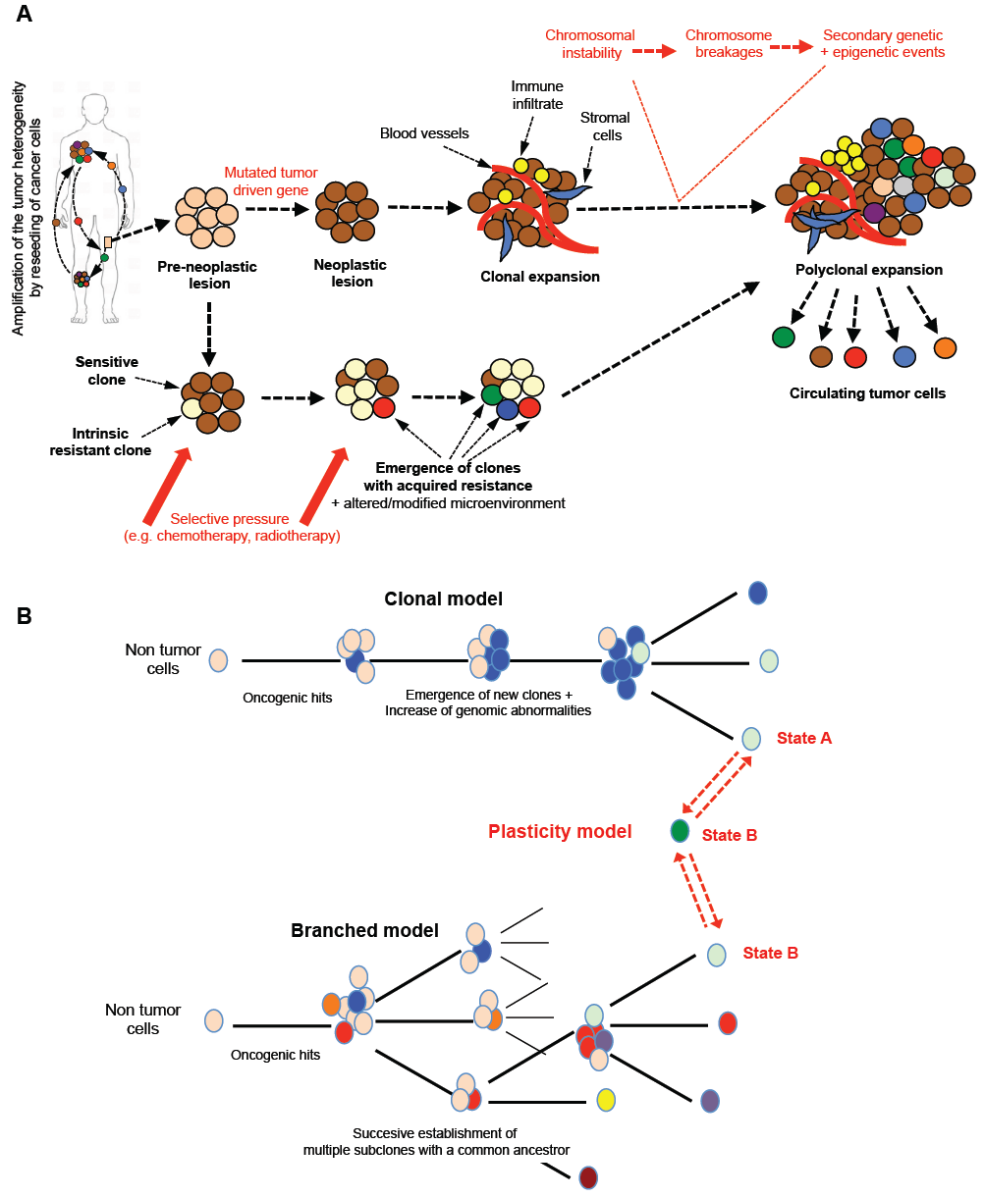
** Tumor models and tissue heterogeneity. A.** From a pre-neoplastic lesion to the development of metastases, the tumor tissue will undergo a marked cellular evolution leading to polyclonal disease. Tumor driven genes appearing in determined normal cells will be responsible for chromosomal instability with numerous chromosome breakages (fusions, deletions, etc) concomitant to secondary genetic and epigenetic events. From the detection of the first oncogenic event, new clones will be formed and will enrich the heterogeneity of the tumor. The pressure of the local micro-environment and/or the therapeutic pressure will enrich the tumor mass in dominant/resistant clones, which will leave the primary tumors to spread to distant organs. Tumor heterogeneity is a property of cancers sustained and amplified by the reseeding of cancer cells from one site to distant foci. **B.** Several models of tumor development have been proposed and may coexist simultaneously in a single tumor mass. Three main models can be described: i) clonal evolution of an initial cancer cell in which subsequent genomic abnormalities occur will lead progressively (in a linear manner) to the emergence of new clones; ii) the various oncogenic events can also lead to the establishment of multiple subclones with common ancestors; this type of model is called the “branched model”; iii) more recently, both models have been completed by the “plasticity model” directly related to the plasticity property of cancer cells. One cancer cell can evolve between two phenotypic states, A/B, linked to various functional states explaining the co-existence and equilibrium of a mixed population expressing a large panel of fusion genes or/and cluster of differentiation and contributing to the polyclonal expansion and heterogeneity of the tumors. This type of mechanism increases the chance of survival for a cancer cell by upmodulating its adaptability to the micro-environment in a permanent manner.

Cancer is characterized by the development and growth of abnormal cell populations (e.g. mutations, altered proliferation and/or differentiation). As DNA is the only cell component that can accumulate and transmit changes throughout life, it has been accepted that the carcinogenesis process requires the progressive accumulation of multiple DNA modifications 2. The current, generally-accepted model for carcinogenesis is the somatic mutation or clonal evolution theory (Table A) 3, 4. Although it is the prevailing model for carcinogenesis, it has been challenged by several lines of evidence 1, 2, 5, 6. The main alternative model focuses on the concept of asymmetric division initially observed in healthy tissue renewal 1, 2, 5, 6. This process is defined as a biological process in which a single cell generates two daughter cells with two distinct destinies: one undifferentiated cell - a stem cell - expressing stemness markers (e.g. Oct4, nano, sox2) and in charge of the self-tissue renewal, and one committed to a specific form of differentiation 14. A similar process has been proposed in cancer leading to the maintenance of cancer-like “stem-cells” harboring the mutated driven gene and participating in the dynamic enrichment of the spatial and temporal heterogeneity of cancer cells 1,5,15,16. Regardless of the carcinogenesis model, mutations in the tumor driven gene expressed by cells from pre-neoplastic lesions (e.g. *TP53*, *Rb*) lead to the formation of neoplastic foci characterized at the early stage by monoclonal expansions of mutated cells. This type of mutation results in genomic instabilities characterized by a high sensitivity to chromosome breakages and consequently a new series of mutations, deletions, and amplifications 17 (Figure 2A). Random chromosome breakages and secondary genetic events clearly contribute to the development of cancer cells with a new genotype and phenotype, and then to the polyclonal expansion stage of the disease. Epigenetic modifications complete the framework of heterogeneity mapping. As revealed by genetically homogeneous cell lines, intercellular epigenetic alterations (e.g. DNA methylation, miRNA expression, etc) strengthen tumor heterogeneity and the drug response 5,18,19. Genetically- and epigenetically-modified cells are prone to migrate from their primary site. In the natural history of cancer, the diffusion of cancer cells from a primary tumor to a secondary distant organ is frequently observed. Interestingly, several authors have demonstrated the migration of prostate cancer cells from metastastic foci to seed new, distant locations or pre-existing lesions, and consequently permanently enrich the heterogeneity of the tumor 20, 21. Thus, the bidirectional seeding between different tumor sites plays a part in enriching tumor heterogeneity.

Similarly to embryonic cells, cancer cells are not blocked in a defined state and adapt permanently their phenotype under the microenvironment and therapeutic pressure. Indeed, phenotypic and functional plasticity is a common mechanism observed during embryonic development 22. Blastocyst cells are composed of various cell types including stem cells, lineage-committed progenitors and differentiated cells linked by hierarchical relationships that make possible the formation of all the organs of the future embryo/fetus and also the extra-embryonic annex from the pre-implantation step to the final gestation. All of these cells can switch between different cell states thanks to a high degree of plasticity. Similarly, cancer cells exhibit marked plasticity as illustrated by the epithelial-mesenchymal transition (EMT) in which cancer cells progressively acquire a mesenchymal phenotype and lose their epithelial properties, thus leading to the metastatic process. In the opposite direction, at the metastatic location, cancer cells undergo mesenchymal-epithelial transition (MET) and re-acquire their epithelial characteristics 23. Inoculation into immunocompetent mice of pure EpCAM^+^ or EpCAM^-^ breast cancer cells, sorted by flow cytometry, led to the detection of mixed EpCAM^+^/EpCAM^-^ cells in the blood stream after a couple of days. This simple experiment perfectly illustrates the extreme plasticity of cancer cells 24 (Figure 2B). The notion of plasticity should be extended to any subtle equilibrium making possible the functional orchestration of various cell populations. Recently, Franzetti *et al.* studied the impact of EWSR1/FLi1 expression on the functional behavior of Ewing sarcoma cells 25. They observed that cancer cells fluctuated over time from low to high EWSR1/FLi1 expression in a reversible process, and the low expression phenotype was correlated with a metastatic profile (e.g. high propensity to migrate and invade). Both cell populations can co-exist in patient samples and EWSR1/FLi1^Low^ contribute to the maintenance of tumor growth based on ESWR1/FL1 re-expression. Their manuscript illustrates a new model of phenotypic plasticity and gives evidence of the functional impact of this dynamic phenotypic fluctuation associated with a dominant oncogene.

However, the therapeutic pressure plays a significant role in the selective amplification of tumor heterogeneity and contributes to emergence of specific dominant clones driving the tumor heterogeneity 26. A tumor mass is composed of a panel of cancer cells with sensitivity or innate resistance to a specific drug or specific therapeutic intervention 29 (Figure 2). Drug resistant clones are then preferentially chosen and in turn selectively modify the tissue heterogeneity. Therapeutic selective pressure is also responsible for acquired resistance mechanisms resulting in the dynamic emergence of new cancer cell clones leading to dynamic heterogeneity. The notion of drug resistance is also related to persister cells observed in cancer and in micro-organisms 5. Persisters are low proliferating cells with a stem-like profile and immune tolerant activities. Overall, the literature demonstrates that tumor heterogeneity becomes an obstacle to determining the appropriate therapeutics in oncology because of the temporal instability of tumor tissue organization. The dynamic evolution of dominant clones and persister cells fuel the tumor heterogeneity which is enriched by a heterogeneous local micro-environment.

### Heterogeneity of the tumor micro-environment: the functional relationship of tumor heterogeneity

**Figure 3:**
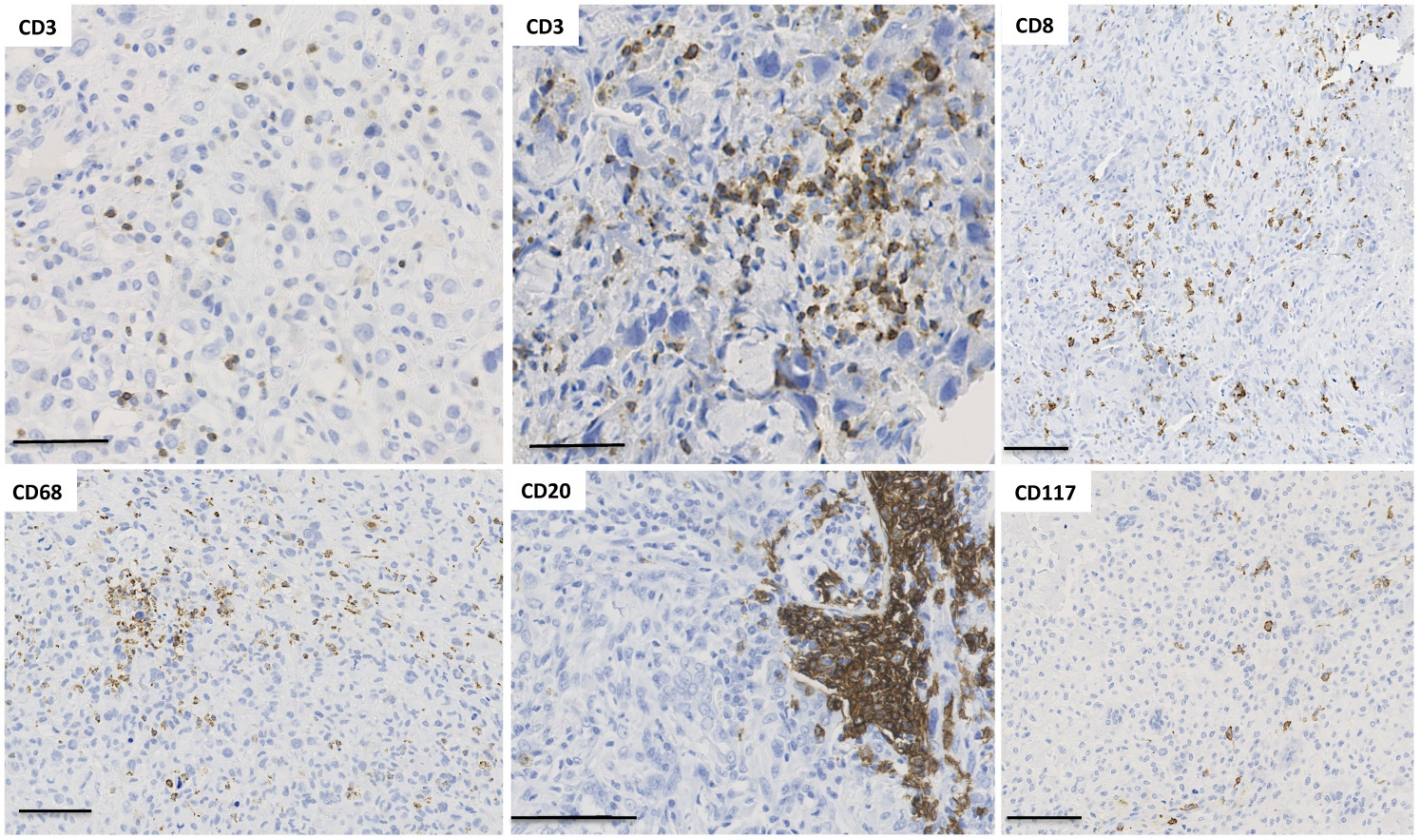
** Spatial immunological heterogeneity of tumor tissue.** Illustration of the heterogeneity of immune infiltrates associated with human osteosarcoma (cohort previously published in 137). Numerous immune cell subtypes invade osteosarcoma tissues during tumor development. Interestingly, their spatial distribution shows a high heterogeneity across the tumor tissue, with CD3^+^ T lymphocytes organized in a diffuse infiltrate as well as small clusters. The localization of CD8^+^ T cells is diffuse with one area without any infiltrated cells. Macrophages exhibit similar distribution to CD3 and the number of CD20^+^ B lymphocytes is relatively low but B cells are sometimes organized in pseudo-nodules. CD117^+^ mastocytes are also observed as diffuse infiltrate in a specific area.

As described above, from a clonal disease, the successive mutations in tumor cells play a part in temporal heterogeneity and the establishment of a very complex polyclonal oncogenic disease. In addition to the heterogeneous populations of neoplastic cells, tumor bulk is composed of non-neoplastic resident cells, the extracellular matrix 7-10, fibroblasts (called cancer-associated fibroblast) 7-10, blood vessels 7-10 and immune cells 7-10 that together form the tumor micro-environment (TME) (Figure 3). MALDI imaging mass spectrometry makes it possible to visualize tumor heterogeneity at the protein level 7-10. Extracellular matrix is a key factor related to metastasis efficiency, controlling collective cell invasiveness 7-10. This observation is related to the diversity of cancer-associated fibroblasts (CAF) 7-10. Indeed, Costa *et al.* identified four subsets of CAF in breast cancer with specific distinct functional properties. In triple negative breast cancers, one of them, called CAF-S1, promotes an immune tolerant environment and stimulates T lymphocytes toward an immunosuppressive phenotype (CD25^high^ FOXP3^high^). The second, called CAF-S4, increases the T cells' regulatory property to inhibit T effector proliferation. Consequently, the local accumulation of CAF-S1 then contributes to tumor heterogeneity and to local immunosuppression observed in triple negative breast cancers. Such immunoregulation is tightly controlled by the production of local immunocytokinic signals leading to a balance between inflammatory and immunosuppressive effectors 7-10. The functional impact of CAF on local tumor immunity is directly linked to the spatial and temporal heterogeneity of T lymphocytes and macrophages observed in numerous types of cancer [31-33[7-10]. Interestingly, resident lymphocytes seem pre-adapted to specific tissues and can adapt to wherever they migrate [34[7-10]. As a consequence of local immune regulation, endothelial cells exhibit several phenotypic features and lead to the formation of specific tumor vasculature 7-10. Interestingly, Hamilton *et al.* revealed that CTCs are competent to modulate tumor associated macrophages in order to increase invasiveness of cancer cells, angiogenesis and immunosuppression 7-10. The quality (e.g. topographic localisation) and quantity of the immune infltrates into tumor tissues have strong impacts on patients' clinical outcomes. New technologies such as multispectral imaging will allow to obtain a precise analysis of these infiltrates and may lead to a better patient stratification 7-10. All components of the tumor microenvironment then play a part in generating more tumor variability, as well as being highly heterogeneous and crucial for determining the development of cancer 7-10. After the tumor excision and the initiation of the therapy, the key challenging question remains the follow up of the tumor heterogeneity in absence of tumor tissue access? Do CTCs reflect the tumor heterogeneity?

### The characterization of circulating tumor cells for predicting tumor heterogeneity

Tumor heterogeneity has challenged the potential benefits of precision medicine. Current methods used to analyze tumor masses and further therapeutic design are based on a global overview of the characteristics of cancer tissues, corresponding to an average picture of all the tumor clones and their micro-environment without considering their diversity 11. This variability limits the prognosis and predictive power of a biomarker. Moreover, differences in the evolution of tumor cells and micro-environments at the metastatic sites question the utility of the biomarkers previously identified in the primary tumor for treating metastatic disease [12, 13]]. Monitoring changes in cell populations during disease progression and treatment will improve both cancer diagnosis and therapeutic design. Current protocols to check the consistency of the biomarkers, establish diagnoses and define treatment are based on very small biopsies (e.g. needle biopsies) of the primary tumor and metastatic sites. The main limitation is that most metastases are difficult to access, and biopsies are invasive, inconvenient, costly and do not make possible longitudinal follow-up of tumor heterogeneity. To overcome these problems, detecting and characterizing heterogeneity in CTCs could be a good alternative and opportunity.

### CTC characteristics as a snapshot of tumor heterogeneity

In the last decade, numerous clinical studies revealed the link between CTC numbers and metastatic prognosis 49, 50. Similarly, the phenotypic properties of CTCs can be related to overall patient survival 51. In the most recent meta-analyses published, the authors found a correlation between CTC count and clinical progression. Recently, by combining more than 20 published studies, CTC count was shown to be an independent and quantitative prognostic factor in patients suffering from early breast cancer 52. At the protein level, there are some discrepancies depending on the series analyzed. In breast cancer for instance, HER2 expression between initial tumor tissues and corresponding CTCs are contradictory, with a concordance of around 90% for some of them 52,53, inconsistency for others 54 or fluctuating variations in the course of the disease 55. Similar to EpCAM, the high plasticity of cancer cells results in variations in HER2 expression and reflects a snapshot of tumor heterogeneity at a specific time point. However, several studies support the idea that cellular heterogeneity in CTCs reflects the spectrum of mutations in the primary tumor and metastatic lesions. Mohamed Suhaimi *et al.* detected a high concordant mutation list in CTCs and tissues (e.g. KRAS and BRAF markers), predicting the outcome of anti-EGFR therapy in colorectal cancer patients 14. Bingham *et al.* showed that CTCs were representative of the entire spectrum of mutations present in the primary tumor and distal metastases for patients with breast cancer. Their findings suggested the utility of CTCs for identifying targetable mutations and for being used as a biomarker to reveal cell populations sensitive to current or previous therapies 15.

Despite these promising results, the existence of heterogeneity in the CTC compartment has been demonstrated, as shown by Pestrin *et al.*, who observed variability in the mutational status of the PIK3CA gene in breast cancer patients. The PIK3CA mutation profile has prognostic significance and is potentially predictive for the response to agents targeting the PI3K pathway 8 Similarly, Pailler *et al.* observed considerable heterogeneity in ROS1-gene abnormalities in CTCs from non-small cell lung cancer (NSCLC), which could explain the mechanism by which tumor cells can escape sensitivity to ROS1-inhibitor therapy 16. Furthermore, it has also been demonstrated that CTC profiles evolve as the disease progresses, illustrating the temporal heterogeneity of cancer diseases. Tsao *et al.* then mapped the phenotypic evolution of melanoma CTCs and detected the presence of drug-resistant clones harboring different molecular signatures of potential clinical value 17. The discordance observed between CTCs and tumor heterogeneity may be explained by the dynamic heterogeneity of the CTCs 61, 62. Primary tumors as well as metastatic foci are permanently reorganized, resulting dynamically in the differentiation and release of new cancer cell clones into the bloodstream, explaining the partial genomic overlap of CTCs and tumor foci at a specific time point. Consequently, even CTCs cannot fully reflect tumor heterogeneity: they are the mirror and snapshot of the disease's progression as well as the clonal evolution of the tumor foci. Overall, the data currently available has pointed out the major advantages of CTC investigation at the single cell level, possibly representing the most accurate strategy for determining the temporal heterogeneity of the disease.

Single CTC analysis could effectively reveal the high heterogeneity, stochastic changes, and driver mutations in cancer cell populations in order to detect drug resistance, and develop new personalized therapeutic strategies as well as their use as prediction markers. De Luca *et al.* validated a protocol to assess the clonal evolution of metastatic breast cancer, based on single CTC analysis by next generation sequencing (NGS), suitable for the development of new therapeutic strategies in precision medicine 18. Miyamoto *et al.* analyzed single-cell RNA-sequencing (RNA-Seq) profiles in prostate cancer and concluded that there was CTC heterogeneity in signaling pathways that could contribute to treatment failure 19. Pailler *et al.* investigated ALK-copy number gains (CNG) in individual CTCs by filter-adapted fluorescent *in situ* hybridization (FA-FISH) and reported a significant association between the dynamic evolution of the numbers of ALK-CNG and progression-free survival (PFS) in NSCLC patients treated with crizotinib, an ALK/ROS1 inhibitor 20. Paolillo *et al*. found ESR1 mutations in single CTCs from metastatic breast cancer patients associated with endocrine therapy resistance 21. As mentioned above, the tumor cell component is not the only source of heterogeneity. TME elements present high variability that can be assessed by single-cell analysis. Tirosh *et al.* presented an extensive study of the heterogeneity associated with the components that shape the melanoma micro-environment, assessed by single-cell RNA-seq. The authors discovered different micro- environments associated with distinct malignant cell profiles that could be used as prognostic markers 22.

#### CTC cluster enrich CTC heterogeneity and have increased metastatic potential

In addition to single CTCs detectable into the bloodstream, CTCs can be observed in clusters composed by cancer cells and/or in association with non-malignant cells. Recent findings suggest that CTC clusters may have a greater contribution to the metastatic process for mechanical and immune features. Indeed, Au *et al.* demonstrated that cluster CTCs are able to reorganize into single-file chain-like geometries in a rapid and reversible manner with reduced hydrodynamic resistance. Consequently, the progression of cluster CTCs through capillaries is slowed down and cancer cell clusters can traverse easily thin constrictions for extravasation and migration into distant organs 23-25. CTC clusters are rare compared to single CTCs, do not come from intravascular aggregations and arise from oligoclonal cancer cell groupings (called homotypic CTC clusters) reinforcing the CTC heterogeneity 23-25. Heterotypic CTC clusters have been also observed in patient samples. In that cases, CTC clusters are not only composed by tumor cell clones but are associated with tissue-derived macrophages 23-25, fibroblasts 23-25 or neutrophils 23-25. Non-malignant cells escorting CTCs may strengthen the biomechanical properties of CTC clusters and may improve their survival. Very recently, Szczerba *et al.* demonstrated that the association between neutrophils and CTCs drove cell cycle progression within blood and increased the metastatic potential of CTCs in breast cancer patients 23-25.

However, heterotypic CTC clusters have never been associated with circulating lymphocytes. What could be the relationship between T lymphocytes and CTCs? A close relationship between immune cells and cancer cells has been described through the immune checkpoints 23-25. Cancer cells can express programmed death-ligand (PDL-1) which binds to PD-1 and/or B7/1 expressed at the cell surface of T lymphocytes. This binding results in the inhibition of downstream signaling and in the decrease of T cell proliferation and an increase of their apoptosis. Recently, Mishra *et al.* compared the dialog between immune cells and CTCs isolated from non-metastatic and metastatic cell lines inoculated in syngeneic immune-competent mice 23-25. In this model, the metastatic cell line exhibited a significant highrer expression of PDL-1 compared to the non-metastatic cell line and inoculation of activated immune cells had no impact on CTCs established from metastatic cell line in contrast to CTCs produced from non-metastatic cell line. PDL-1 was found to correlate with histological tumor grading and is frequently expressed by CTCs 23-25. Since PDL-1 is a molecular regulator of regulatory T lymphocytes, a CTC immune escape mediated by PDL-1 can be hypothesized 23-25. In such context, T lymphocytes may indirectly contribute to the tumor CTC heterogeneity by selecting specific dominant cancer cell clones that escape to the immune surveillance and lead to the release of specific CTCs into the bloodstream. To illustrate this purpose, Sun *et al.* studied a series of non-small cell lung cancer patients 23-25. They observed that the number of CTCs were positively associated with the metastatic process and negatively associated with the level of circulating T lymphocytes. CTC cluster thanks the interactions between cancer cells and non-malignant cells contribute to the CTC survival, proliferation, immune escape and drug-resistance 23-25.

### Brief overview of the most significant breakthrough technologies for deciphering single-cell characteristics

**Figure 4:**
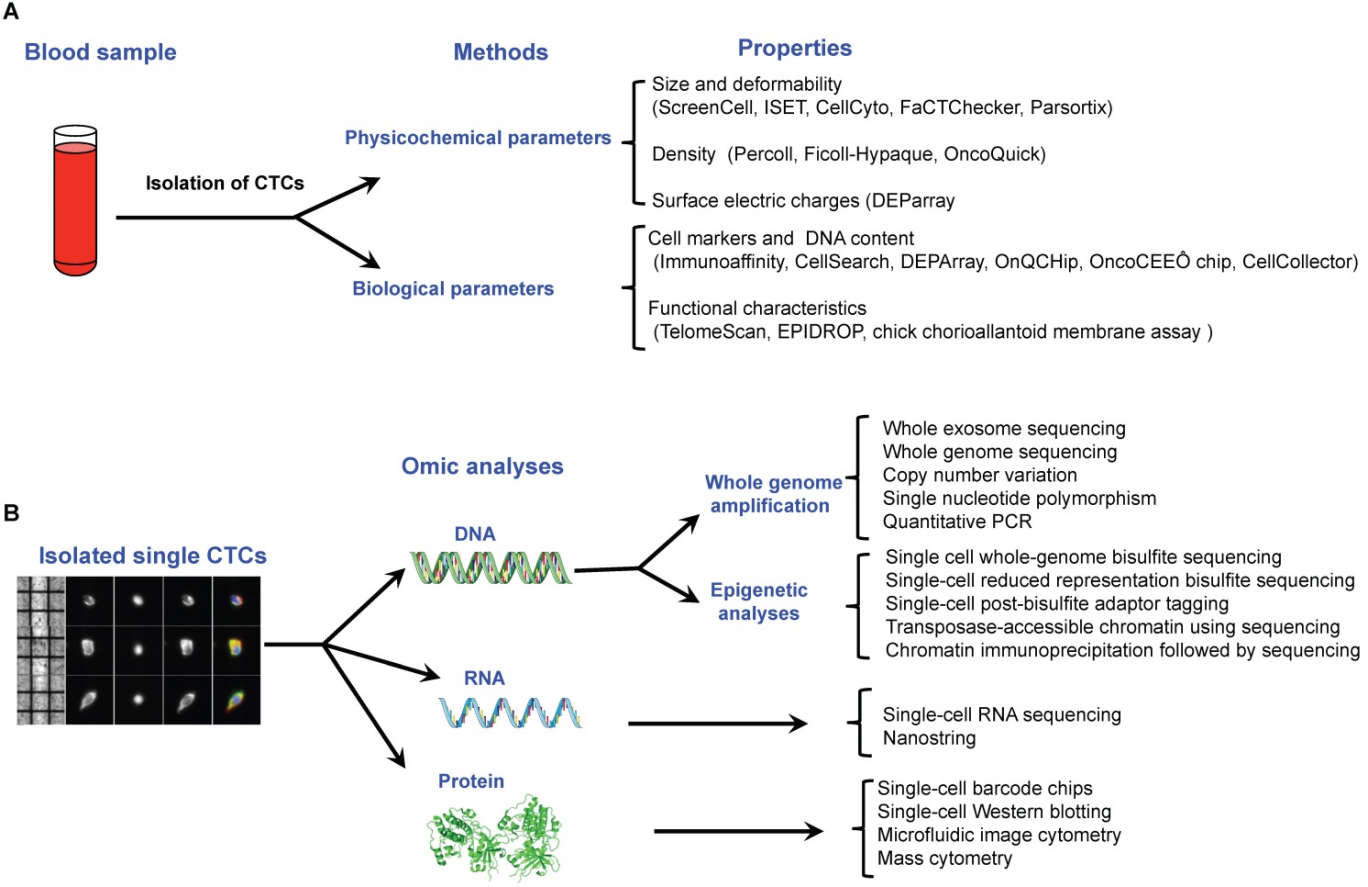
** Recent technological approaches used for isolating and characterising circulating tumor cells. A.** Isolation of single CTCs is based on a two steps method including a pre-enrichment step followed by an isolation approach. All of these methods are related to the physicochemical or biological properties of CTCs. **B.** Single CTCs can be characterized by omic methods at the DNA, RNA and protein levels.

CTCs are rare cell events in blood with a frequency of around one tumor cell for 10^6^-10^8^ normal blood cells. Consequently, before single CTC analysis can take place, the first step is to enrich and isolate CTCs. Multiple technologies have been described to do this in individual CTCs, based on the different properties that distinguish them from surrounding normal hematopoietic cells, including biological properties (cell surface protein expression, viability, invasive capacity) and physical properties (size, density, electric charges, deformability) reviewed previously 23-25 (Figure 4A). Once single cells have been isolated, downstream analyses can be performed depending on the molecular components assessed: DNA, RNA or protein (Figure 4B).

#### Single-cell DNA analysis

Studying genetic variability in single cells has stimulated the development of several high throughput sensitive methods for detecting large patterns of mutations including target-specific amplification using PCR, as a means of querying specific loci of interest 26, and the next generation of sequencing approaches: whole exome sequencing (WES), 27 and whole genome sequencing (WGS) 28 to obtain information regarding the complete exome and genome respectively. As the amount of DNA that can be extracted from a single CTC is limited, an initial step involving whole genome amplification (WGA) is required. Depending on the downstream application, the WGA method selected can differ. Interestingly, recent studies compared different WGA methods and revealed that that multiple displacement amplification (MDA) methods are better suited for single nucleotide polymorphism (SNP) detection, while PCR-based methods are the better option for copy number variant (CNV) detection 29. The authors suggested that AMPLI1 or MALBAC should be used in favor of the REPLi-G or PicoPlex kits when high target coverage is required 30. They also found higher sensitivity of DOPlify and Picoseq for detecting 100% of CNVs than Ampli-1 and REPLI-g 31. After genome amplification, the type of genomic investigation must be defined according to the objective of the study. For instance, Polzer *et al.* used a qPCR assay to analyze the variability of HER2 and PIK3CA in breast cancer single CTCs. Their findings demonstrated that assessing the heterogeneity for these two markers may uncover tumor evolution mechanisms useful for personalized therapy decisions 32. In another study, Janiszewska *et al.* assessed single-nucleotide PIK3CA mutations and HER2 copy number alterations in single cells in formalin-fixed paraffin-embedded breast tumor samples using the STAR-FISH (specific-to-allele PCR-FISH) methodology. This analysis proved to be useful for predicting clinical outcomes in breast cancer patients subjected to neo-adjuvant chemotherapy followed by adjuvant therapy with trastuzumab 33.

In the last few years, there has been a significant increase in the number of studies that use next generation sequence approaches for unraveling single cell heterogeneity. The study performed by Li* et al.* identified 4 new driver mutations in renal cell carcinoma stem cells using WES that constitute important prognostic factors and therapeutic targets 34. Liu *et al.* combined multi-region WES and single-cell WGS to examine the intra-tumor heterogeneity of rectal tumors. Their results suggest a specific architecture for each tumor related to different diagnoses, prognoses and drug responses 35. Sequencing single CTC genomes and exomes provides considerable amounts of information, but also faces several technical challenges, in addition to difficulties with CTC capture 23. The key limitations of single-cell sequencing using WGA are low coverage of the human genome and the consistency of the coverage between single cells 36. This issue could be solved by third-generation sequencing technologies, such as the Pacific Bioscience system 37 and Nanopore sequencing 38. The second challenge is data analysis, as currently the bioinformatics tools used for single-cell sequencing were initially developed and adapted for bulk cell sequencing. New computational and statistical methods have been developed to meet the requirements of single-cell analysis to reduce these biases and address biological and clinical questions more accurately 39-41.

#### Single-cell epigenetic analysis

Robust technologies have been developed for mapping epigenetic marks in single cells. Bisulfite conversion followed by sequencing (BS-seq) is considered the gold-standard method for single base resolution and absolute quantification of DNA methylation levels 39-41. Farlik *et al.* described a whole-genome bisulfite sequencing (WGBS) assay that makes it possible to analyze heterogeneous DNA methylation patterns in single cells (scWGBS) and compared it with the other two methods: single-cell reduced representation bisulfite sequencing (scRRBS) and single-cell post-bisulfite adaptor tagging (scPBAT) 42. The authors concluded that scWGBS is the method of choice for analyzing large numbers of single cells at low sequencing coverage, scRRBS is useful for comparing CpG islands across single cells, and scPBAT is best suited for deep sequencing of single cells with maximum coverage 43. For mapping histone marks at the single cell level, chromatin immunoprecipitation followed by sequencing (ChIP-seq) has recently been implemented. Rotem *et al.* used this methodology to investigate the cell-to-cell variability of different types of regulatory elements and they confirmed its suitability for revealing aspects of epigenetic heterogeneity not captured by transcriptional analysis alone 44. To assess the spatial organization of chromosomes, Kind *et al.* successfully modified the DamID method 45. The heterogeneity of chromatin structure in single cells can be monitored by assay for transposase- accessible chromatin using sequencing (ATAC-seq) 46 and DNase I hypersensitive site sequencing (DNase-seq) 47. Nowadays, it is also possible to assess variability in the 3D structure of chromosomes at the single cell level using a HiC-based method 48. Other methodologies that have been developed to analyze epigenetic changes in bulk samples can potentially be adapted to single cell analysis 49. Despite the fact that analyzing epigenetic state in CTCs is still in its infancy, various studies have already been published in the field. Pixberg *et al.* analyzed the epigenetic status of the genes associated with epithelial mesenchymal transition (EMT) in individual breast cancer CTCs, and explored potential intra- and inter-patient heterogeneity using the agarose-embedded bisulfite sequencing (AEBS) protocol. They found heterogeneous methylation patterns in CTCs with clear infrequent hypermethylation at key promoters of the inhibitor genes of the EMT, suggesting that both epithelial and mesenchymal CTCs can contribute equally to the metastatic process 50.

#### Single-cell RNA analysis

Single-cell RNA sequencing (scRNAs) is the method selected if the aim of the study is to explore transcriptome heterogeneity 51. Interestingly, Ziegenhain *et al.* performed a comparative analysis of the most prominent scRNA-seq methods and identified Drop-seq as the best method for analyzing the transcriptome of large numbers of cells with low sequencing depth, SCRB-seq and MARS-seq are preferable for the transcriptome of fewer cells, and Smart-seq2 would be the appropriate method of choice when annotating the transcriptome of very small quantities of cells 52. All the data generated after scRNAs should be processed to avoid false-positives due to nonlinear amplification, false-negative allelic drop-out due to amplification bias, non-uniform coverage, and noise that arises during single-cell transcript amplification. Specific computational models have been specifically developed to address these issues 53.

Numerous studies show extensive use of scRNAs for unraveling CTC heterogeneity. Patel *et al.* reported the heterogeneity of single glioblastoma CTCs and the existence of high variability in signaling molecules relevant to the targeted therapy, a wide spectrum of stemness and differentiation states, variable proliferative capacity and expression of quiescence markers, all of which were related to the success or failure of therapeutic strategies 54. Chung *et al.* used scRNA to characterize heterogeneity in tumor cells and TME components (mainly immune cells) in breast cancer. The authors identified various signatures in both compartments related to tumor development and the response to cancer therapy 55. Despite the multiple advantages conferred by single- cell RNAseq, it also presents certain limitations that need to be resolved. The main ones are that RNA losses have to be kept to a minimum during cDNA conversion, and that the amplification should provide enough DNA for sequencing without too much quantitative bias or altering of the original picture of the cells' transcriptomic profile. Moreover, scRNA-seq methods use an oligo-dT primer that specifically captures only polyadenylated RNA, avoiding the unwanted amplification of tRNA and rRNA. However, it represents a problem for the non-polyadenylated RNAs such as long non-coding RNA and microRNAs that have been shown to play important roles in cancer 56, 57. Some commercial kits have been developed to overcome the poli(A) tail restriction 58. Another challenge is the low signal-to-noise ratio of single-cell RNA-seq technologies. It is thus important that cell isolation, library preparation, and other automated workflows be as standardized as possible to minimize any bias introduced by human error 59. In this regard, Suzuki *et al.* proposed the use of standard cell lines in future quality controls 60. Finally, many scRNA-seq analyses are still performed using methods originally developed for bulk RNA-seq even if their adaptability to single-cell transcriptomics is unclear 61. As the reliability of the bioinformatic method directly determines the accuracy of the experimental results, it is important to develop bioinformatics tools specific to the analysis of single-cell RNA-seq data, such as the two very recent methods developed by Wu *et al.* 62 and Miao *et al.* 63.

#### Single-cell proteomic analysis

Recent progress in microfluidic technologies and mass spectrometric approaches have led to new single-cell proteomics studies that could be performed with greater sensitivity and specificity. The micro- engraving technique, single-cell barcode chips (SCBCs) and single-cell Western blotting (scWB) are the microfluidic platforms providing the most advanced capabilities 64. The latest version of microfluidic image cytometry (MIC) makes it possible to analyze the heterogeneous expression of up to 90 proteins in each single cell 65. Another technology is CyTOF, a mass cytometry platform that has been developed to assess phenotypic heterogeneity at the single cell level. It can simultaneously image the localization and modifications of 34 proteins (and potentially up to 100) in each cell at subcellular resolution 66. Sinkala *et al.* validated the clinical utility of proteomics in single cells by using scWB, and analyzed variability in metastatic breast cancer cells. They observed high heterogeneity in the expression of 8 key proteins related to breast cancer progression 67.

## Conclusions and perspectives

Repeatable, minimally-invasive and cost-effective approaches for real time assessment of relevant biomarkers and monitoring cancer therapies in the bloodstream have been developed to overcome the intrinsic limitations of primary tumor and metastasis biopsies. This field of investigation has been termed “liquid biopsy” and includes circulating tumor cells (CTCs), exosomes, circulating cell-free DNA (cfDNA), miRNAs and proteins. CTC characteristics can be considered to be a snapshot of overall tumor bulk (primary tumor and metastases). Compared to other liquid biopsies, CTCs are a little bit more laborious to obtain but can be analyzed at the DNA, RNA, and protein level, as well as with regard to their functional cellular characteristics as a means of providing information that relates to the whole cell 67, 68.

Pooling the cells might provide different results and could mask clinically relevant rare mutations 18. A major question emerges regarding the number of CTCs that need to be analyzed in order to capture the overall profile of the dominant disease driving the (sub)clones in a patient suffering from widespread metastatic disease. Gao *et al.* conducted a study on this subject and concluded that around 20-40 single cells are required to detect the main subclones with 95% power 69. Despite promising results, showing a high concordance between paired CTCs and primary tumors or metastatic sites 15, 70, many other studies found discordant results between the mutational status of CTCs and those of the corresponding primary tissue or metastasis 71-73. Like tumor tissues, CTCs are in fact heterogeneous in all the cancer types analyzed 57,60, 131-143 (Table 1). Very recently, Sun *et al.* demonstrated the existence of specific CTC territories, marking the spatial heterogeneity of CTCs. These authors compared the EMT status of CTCs isolated from various vascular territories and observed surprisingly high heterogeneity depending on their location 138 and hypothesized that spatial CTC heterogeneity could impact both the recurrence of the disease and the metastatic process. This could be explained by the fact that CTCs reflect the dynamic evolution of the advanced stages of cancer more closely than the primary tumor (temporal heterogeneity) 18.

EMT biomarkers (e.g. TWIST1, SNAI1/2, N-cadherin, vimentin) are differentially expressed in CTCs 144-146. EMT was shown to enhance metastatic properties of tumor cells. Markiewicz* et al.* analyzed the link between the detection of breast cancer CTCs with a mesenchymal phenotype and EMT status of primary tumors 147. In their series, mesenchymal phenotype of CTCs was more frequent in primary tumors with E-cadherin loss compared to those with normal E-cadherin expression. However, EMT status of matched samples at different stages of dissemination was frequently discordant, especially for pairs associating CTCs. In more of 500 breast cancer patients, CTCs were detected in only 19% of blood samples 148. These authors identified a subset of primary breast cancer patients with EMT (29%) and stem cell (14%) phenotype and they did find any correlation between these markers and other prognostic clinical markers. Similarly, it has been shown that around 30% of metastatic prostate cancer patients had no detectable EpCAM^+^ CTCs 149. More recently, Lowes *et al.* studied the EMT process and CTC release in pre-clinical models of prostate cancer 150. They confirmed that that the method used for isolating CTCs is crucial and that CellSearch®-based assay used in their study failed to detect around 40-50% of CTCs with mesenchymal phenotype. Overall, these studies confirmed the high plasticity of cancer cells and demonstrated that the current methods used for detecting/isolating all subtypes of CTCs which undergo EMT are not efficient enough. Novel technological approaches are required to better follow the metastatic disease.

Nowadays, most of the studies published focus on revealing cell-to-cell differences at the DNA and RNA level. For overall understanding of single-cell heterogeneity, future studies should focus on the combination of different multi-omic assays on the same cell, such as the study performed by Hou *et al*. in which the authors used a single-cell triple omics sequencing technique called scTrio-seq which links the complex contribution of genomic and epigenomic heterogeneities to transcriptomic heterogeneity within a population of cells 151. After conquering the barrier of multi-omics analysis for single cells, the final challenge will be temporal and spatial measurement of the molecular profile in a single cell. New technologies should not only solve the problem of the existing analysis methods which characterize only a snapshot profile of CTCs, but also provide real-time dynamics to measure patient status and then to follow the heterogeneity of the disease. The primary aspect of this new technology is *in vivo* monitoring and analysis of single CTCs, as has been shown in different studies 152, 153, but the high cost and lack of sensitivity prevent it from serving as a routine clinical test. In addition, future studies should also consider the importance of micro-environment and immunological elements in the heterogeneity state of cancer cells, as numerous emergent studies have demonstrated their consequences in tumor evolution and therapeutic response 7, 9, 10, 154. In this regard, efforts must be made to clarify the complex interplay among cells within the tumor ecosystem and between functional states in space and time before its translational application 22 (Table B). Understanding tumor heterogeneity is of the utmost importance, as this phenomenon is associated with a decrease in diagnostic precision and is an obstacle for designing appropriate therapeutic strategies. Enumeration and molecular profiling of CTCs may be useful for a better patient stratification. High content analysis of CTCs can give a snapshot of the tumor heterogeneity at a given time and could allow to adapt therapeutic approach all along the treatment. Indeed, CTCs like all cancer cells are highly plastic and can modify their phenotype according the micro-environmental and therapeutic pressure. EGFR-mutated non-small lung cancer is a good illustration of cancer cell plasticity related to drug resistance 22. EGFR-mutated NSCLC is a genetically heterogeneous disease with more than 200 distinct mutations. The identification of the most common L858R mutations-predict sensitivity to EGFR tyrosine kinase inhibitors. However, some patients become progressively resistant to the first line of tyrosine kinase inhibitor by developing new sets of mutations of EGFR illustrating again the plasticity of cancer cells. CTCs collection is weakly invasive and would allow the follow up of EGFR mutation status in order to adapt the therapy “in real time” 155. Genetic/ epigenetic/molecular profiling of CTCs open new era of personalized medicine. Unfortunately, the implementation of single CTCs in clinical practice is still limited because technologies are expensive, time-consuming and require standardization processes. Current protocols should be replaced by new ones that make it possible to obtain results in a short time and thus avoid any delay in treating the disease for cancer patients.

## Figures and Tables

**Table A:** Models of carcinogenesis.

• The somatic mutation or clonal evolution theory is based on DNA changes in oncogenes and tumor-suppressor genes that lead to alterations in cell proliferation and/or cell-cycle arrest and/or cell differentiation and/or inhibition cell death. • The stem cell division theory for cancer suggests that Tumor-Initiating Cells (TICs) are the origin of cancer development. TICs are characterized by their capacity for self-renewal and play a part in the development of the heterogeneous lineages of cancer cells by accumulating successive asymmetric cell divisions. Similar to stem cells, TICs carry individual DNA from zygote to death and therefore hold the DNA long enough to accumulate the alterations required for carcinogenesis.

**Table 1:** Recent studies analyzing the heterogeneity of circulating tumor cells (CTCs) at the single cell level.

Analytical methods	Isolation methods	Number of patients	Clinical relevance	Reference
Allele-specific PCR	Size-based microsieve technology	44	Analysis of KRAS and BRAF heterogeneity analyzed in CTCs can predict outcomes of anti‐EGFR therapy in colorectal cancer patients.	54
Foundation One™	CellSearch followed by single-cell isolation by DEPArray Markers used : EpCAM^+/-^, CD45^-^, DAPI	32	CTC analysis can be used to identify targetable mutations, and as a biomarker to reveal the sensitivity to therapy of different breast cancer cell populations	55
PI3KCA Sanger sequencing	CellSearch followed by single-cell isolation by DEPArray Markers used : EpCAM^+/-^, CD45^-^, DAPI	39	Detection of variability in PIK3CA gene mutational status in single CTCs isolated from breast cancer patients. PIK3CA mutations have prognostic significance and are potentially predictive for response to agents targeting the PI3K pathway	56
Filter-adapted-fluorescence *in situ* hybridization (FA-FISH)	Filtration, Isolation by size of epithelial tumor cells (ISET)	8	Heterogeneity of ROS1-gene abnormalities in CTCs from non-small cell lung carcinoma could explain the tumor cells' resistance to ROS1-inhibitor therapy	57
Antibody-conjugated and surface-enhanced Raman spectroscopy (SERS)	No CTC isolation, detection of CTCs in blood samples	10	Detection of cell heterogeneity in CTC drug-resistant clones with potential clinical value for treatment decisions (melanoma)	58
Next Generation Sequencing (NGS)	CellSearch followed by single-cell isolation by DEPArray Markers used : EpCAM^+/-^, CD45^-^, DAPI	4	High intra-tumor heterogeneity in breast single CTCs in genes related to therapeutic response. This can be used to assess the clonal evolution of metastatic breast cancer and further therapeutic intervention based on the mutational status.	61
Single-cell RNA-sequencing (RNA-Seq)	CTC-iChip Markers used : EpCAM^+/-^, CDH11^+/-^, CD45^-^	22	Complex inter-tumor and intra-tumor heterogeneity in drug resistance mechanisms of analyzed prostate single CTCs relates to anti-androgen therapy failure.	62
FA-FISH	Enrichment by ISET, enumeration by CellSearch Marker used: EpCAM^+/-^, CD45^-^	18	Inter-tumor heterogeneity in CTC numbers with ALK-copy number gains has significant association with crizotinib efficacy and progression-free survival (PFS) in non-small cell lung carcinoma.	63
Sanger sequencing	CellSearch Marker used: EpCAM^+/-^, CD45^-^	30	Clonal heterogeneity analysis in single CTCs from metastatic breast cancer patients revealed early ESR1 mutations associated with endocrine therapy resistance. This can be used to predict which patients will benefit from a given therapy.	64
RNA-Seq	Flow cytometry: CD45^-^	19	Detection of intra- and inter-individual heterogeneity in melanoma cells and tumor micro-environment components linked to resistance to targeted therapies.	65

**Table B:** Precision of medicine: the future

• Losing a significant amount of CTCs can be associated with subsequent misinterpretations of heterogeneity, and thus bad clinical decisions. • Future studies should focus on the combination of different multi-omics assays on the same cell to obtain a heterogeneity profile at different molecular levels. • Efforts should be made to implement in clinical practice real-time heterogeneity single CTC analysis to measure patient status at any time in the course of the disease. • Many studies have shown that TME plays an important role in tumor heterogeneity. Upcoming research studies assessing tumor heterogeneity thus need to include the analysis of different components in the TME. • It is mandatory to run clinical trials to clarify the clinical utility of CTC data. • The implementation of single cell heterogeneity analysis in clinical practice is a priority for improving precision medicine. Scientific and clinical communities should concentrate their efforts on solving the problem of high cost and time-consuming technologies. • A social debate is open for the near future regarding the difficulties many patients have with affording the high cost of getting diagnosed by precision medicine technologies.

## Glossary

Circulating tumor cells (CTCs): originate from the primary tumor or metastatic foci and at least some of them are able to invade the surrounding tissue, enter either the lymphatics or the bloodstream, survive in circulation, extravasate into a tissue and finally grow at the new site
Tumor micro-environment (TME): is the cellular environment in which the tumor exists, including surrounding blood vessels, immune cells, fibroblasts, bone marrow-derived inflammatory cells, lymphocytes, signaling molecules and the extracellular matrix. TME and tumors are closely-related and interact constantly. Cells in the micro-environment can affect the growth and evolution of tumor cells, while cancer cells can induce changes in the micro-environment by releasing extracellular signals, promoting tumor angiogenesis and inducing peripheral immune tolerance
Liquid biopsy: non-invasive test performed on blood samples for the capture and analysis of molecules originating from tumors such as CTCs, exosomes, miRNAs, proteins and circulating cell-free DNA. The main potential applications of this technique are: the screening and early detection of cancer; relapse-risk estimation; identification of therapeutic targets for precision medicine; and real-time monitoring of response to therapy and anticipation of emergent therapy resistance
Precision medicine: refers to the adjustment of medical treatment to the individual characteristics of each patient. It does not literally mean the creation of drugs or medical devices that are unique to a patient, but the ability to classify individuals into subpopulations that differ in their susceptibility to a particular disease, in the biology or prognosis of those diseases they may develop, or in their response to a specific treatment. Preventive or therapeutic interventions can then be concentrated on those who will benefit, sparing expense and side effects for those who will not
